# Epicutaneous Immunotherapy Compared with Sublingual Immunotherapy in Mice Sensitized to Pollen (*Phleum pratense*)

**DOI:** 10.5402/2012/375735

**Published:** 2012-02-02

**Authors:** Lucie Mondoulet, Vincent Dioszeghy, Mélanie Ligouis, Véronique Dhelft, Emilie Puteaux, Christophe Dupont, Pierre-Henri Benhamou

**Affiliations:** ^1^DBV Technologies, R&D Department, 92220 Bagneux, France; ^2^Hôpital Necker, Gastroenterology Department, 75014 Paris, France

## Abstract

*Background*. The aim of this study was to compare the efficacy of epicutaneous immunotherapy (EPIT) to sublingual immunotherapy (SLIT) in a model of mice sensitized to *Phleum pratense* pollen. *Methods*. BALB/c mice were sensitized by sub-cutaneous route to pollen protein extract mixed treated for 8 weeks, using sham, EPIT, or SLIT. Measurements involved the serological response and cytokine profile from reactivated splenocytes, plethysmography after aerosol challenge to pollen, cell, and cytokine contents in the bronchoalveolar lavages (BALs). *Results*. After immunotherapy, sIgE was significantly decreased in the treated groups compared to sham (*P* < 0.001), whereas sIgG2a increased with EPIT and SLIT (*P* < 0.001 and *P* < 0.005 versus sham). Reactivated splenocytes secreted higher levels of Th2 cytokines with sham (*P* < 0.01). Penh values were higher in sham than EPIT and SLIT. Eosinophil recruitment in BAL was significantly reduced only by EPIT (*P* < 0.01). *Conclusion*. In this model of mice sensitized to pollen, EPIT was at least as efficient as SLIT.

## 1. Introduction

Specific immunotherapy has been used for almost a century in allergic patients to redirect inappropriate immune responses. The long-term benefits of subcutaneous immunotherapy (SCIT) were demonstrated by Durham et al. in patients with grass pollen sensitization [1]. SCIT is now considered as the reference method by the World Health Organization (WHO) and the gold standard treatment of allergy [2].

 For various reasons, noninvasive routes (e.g., nasal, oral, or sublingual) have been evaluated as alternatives to subcutaneous injections [3], the sublingual route being the most widely used. Many trials have established the safety and efficacy of sublingual immunotherapy (SLIT) [4, 5]. A model of SLIT in mice sensitized to timothy grass allergen (*Phleum pratense*—Ph1p) has been reported [6].

Recently, Senti et al. [7] have proposed a new method of noninvasive immunotherapy using the epicutaneous route in patients sensitized to pollen. This method is a promising alternative to the well-established SCIT and SLIT [3]. In parallel, we communicated encouraging results of epicutaneous immunotherapy (EPIT) in children allergic to cow's milk [8] and published data of EPIT in a mice model using various allergens [9]. The latter were obtained using Viaskin, a new epicutaneous delivery system (EDS).

The purpose of the present study was to compare EPIT to SLIT with regards to the immune response they generate in mice sensitized to timothy grass pollen.

## 2. Materials and Methods

### 2.1. Animals and Study Design (Figure 1)

Three-week-old female BALB/c mice (Charles Rivers, Lyon, France) were purchased and housed under standard animal husbandry conditions. Mice were acclimated for 1 week before immunization. After a phase of sensitization validated by an increase in specific IgE (sIgE), mice were randomly allocated to 3 groups of 10 animals and treated during 8 weeks: (1) EPIT, treated by epicutaneous immunotherapy (EPIT 100 *μ*g), (2) SLIT, treated by sublingual immunotherapy (SLIT 100 *μ*g), or (3) sham treated by empty EDS and solution vehicle used in SLIT. Ten naive mice (not sensitized and not treated) were also included.

Seven days after the end of the treatment, animals were challenged for 3 days with grass pollen aerosol. Animals were then submitted to evaluation of airway hyperresponsiveness (AHR) by plethysmography. Finally, all animals were submitted to a bronchoalveolar lavage (BAL) for cytological and immunological analyses and to a culture of spleen cells for cytokine analysis.

Skin preparations before EDS application or sublingual administrations were all performed under general anesthesia by Ketamine (Imalgen1000, Merial) (100 mg/kg body weight) and Xylazine (Rompun, Bayer) (10 mg/kg body weight). BAL was performed under general anesthesia by intraperitoneal administration of pentobarbital (Nembutal, Sanofi Santé animale, CEVA) (50 mg/kg body weight), and venipuncture was performed under general anesthesia by isoflurane (Isoflurane Belamont, Nicholas Piramal India).

All experiments were performed according to the European Community rules on animal care with permission 92–305 from the French Veterinary Services.

### 2.2. Immunization

Mice were sensitized to pollen by means of three subcutaneous injections at one week interval on the back of the neck with 10 *μ*g of *Phleum pratense* pollen protein extract (ALK, Varennes en Argonne, France) and 1 mg of aluminum hydroxide (Sigma, Saint Quentin Fallavier, France) using a protocol adapted from Wiedermann et al. Blood specific IgEs were quantified 10 days after the last injection to confirm sensitization. Naïve mice received 200 *μ*L of sterile solution containing 0.9% NaCl by subcutaneous injections according to the same scheme.

### 2.3. Treatment

#### 2.3.1. Epicutaneous Immunotherapy (EPIT)

EPIT was performed using an original epicutaneous delivery system (EDS) (Viaskin, DBV Technologies, Paris France) consisting of a central transparent polyethylene membrane (11 mm in diameter) surrounded by a clear double-sided adhesive polypropylene film to maintain the chamber on the skin. Dry powder of pollen extract is maintained on the backing by electrostatic forces. An occlusive chamber is created on the skin that rapidly generates moisture and releases the allergen from its support. The allergen is then absorbed by the skin where it interacts with epidermal immune cells. The use of this technology as diagnosis purpose (Diallertest) was previously published [10].

EDS with 100 *μ*g of pollen protein extract (containing 5 *μ*g of Ph1 p 5) was applied for 48 h to the back of mice, once a week during 8 weeks. Twenty-four (24) hours before application, skin was shaved with an electric clipper and depilatory cream was applied. This technique does not modify the barrier properties of the skin. This was demonstrated in a previous experience by the absence of change in the transepithelial water loss (TEWL), as compared with hairless mice (6.45 ± 1.22* versus *6.63 ± 1.49 g/h/m², ns).

#### 2.3.2. Sublingual Immunotherapy (SLIT)

Once a week during 8 weeks, mice of the SLIT group received 5 *μ*L of a homogeneous preparation of 100 *μ*g of pollen protein extract (containing 5 *μ*g of Ph1 p 5, the optimal SLIT dose described by Brimnes et al. in their murine model [6]) in PBS administered sublingually under the tongue. Similarly, mice of EPIT and Sham groups received 5 *μ*L of phosphate-buffered saline (PBS). To increase their viscosity, the sublingual solutions were prepared with 1.2% of carboxymethylcellulose (CMC) (w/v) (Sigma, Saint Quentin Fallavier, France). To prevent the animals from swallowing the solution (allergen or PBS), weekly administrations were all performed under anesthesia with ketamine and xylazine.

During general anesthesia, the distribution of sublingually applied solutions was investigated with additional mice, as previously described by Brimnes et al. [6] using 1% Evans Blue solution in PBS with CMC. The mice were sacrificed 5 min (*n* = 6), 15 min (*n* = 6), and 30 min (*n* = 6) after the administration of the dye and the distribution evaluated in the sublingual tissue, esophagus, and stomach by visual inspection and extraction of tissue in acetone/Zephiran solution as described by Caster et al. [11] followed by measurement of absorption at 620 nm in microtiter plate using Multiscan Ex plate reader (Thermo Scientific, Cergy-Pontoise, France). Two mice without administration of dye were also evaluated as negative controls. Thirty minutes after sublingual administration, dye was only detectable in the sublingual tissue but not in the esophagus and in the stomach. The absorbance in sublingual tissues decreased from 0.41 (at 5 min), 0.22 (at 15 min) to 0.15 (at 30 min) compared to 0.04 (*P* < 0.001) in control sublingual tissues.

#### 2.3.3. Sham and Control Groups

During the immunotherapy period, the Sham group received both an empty EDS and a sublingual administration of PBS and carboxymethylcellulose (1.2%, w/v) on a weekly basis, following the same procedures as for the EPIT and SLIT groups. The control group was not sensitized and not treated.

### 2.4. Specific IgE, IgG1, and IgG2a in Blood

Blood was collected from the retroorbital venous plexus 10 days after sensitization (D0) and during immunotherapy (D21, D38, D63).

Specific antibodies were quantified using a quantitative ELISA developed in-house according to the 2001 FDA guidelines. Briefly, microtiter plates were coated with 100 *μ*L per well of 5 *μ*g/mL pollen protein extract solution. Serial dilutions of 50 *μ*L of each serum were dispensed per well and incubated for 24 h at +4°C. An anti-mouse IgE, IgG1, or IgG2a antibody labeled with phosphatase alkaline (Serotec, Oxford, England) was used as a tracer. P-nitrophenyl phosphate (pNPP—Sigma, France) was used as the enzyme substrate. Specific IgE, IgG1, and IgG2a were quantified by comparison with concentration-response curves obtained with a total IgE, IgG1, or IgG2a assay performed under identical conditions using a solid phase coated with an anti-mouse IgE, IgG, or IgG2a antibody (Serotec, Oxford, England). The cross-reactivity of secondary antibodies with immunoglobulins was less than 4% for all the antibodies and less than 0.1% for anti-IgG1 and anti-IgG2a antibodies against purified IgE.

### 2.5. Cell Culture

After BAL, mice were sacrificed and spleens teased into a single-cell suspension and washed three times in RPMI-1640 (Dutcher, Brumath, France). After lysis of the red blood cells (180 nM NH_4_Cl, 17 mM Na_2_EDTA) and several washes, splenocytes were resuspended in RPMI supplemented with 10% FCS, 2 mM L-glutamine, 100U penicillin, and 100 *μ*g/mL streptomycin. Cells were counted, adjusted to 3 × 10^6^ cells/mL in each well of a 24-well flat-bottomed culture plate (Nunc), and stimulated by Phl p extract (100 *μ*g/mL) or by its solvent (RPMI supplemented with FCS, L-glutamine, penicillin, and streptomycin). The cells were cultured in presence or not of Phleum pratense extract (100 *μ*g/mL per well) for 72 h at 37°C and 5% CO_2_. Supernatants were then removed and stored at −80°C until further assay.

### 2.6. Cytokine Levels and Cell Composition in Blood, BAL Fluid, and Cell Culture Supernatants

Blood samples for cytokine analyses were collected in anesthetized mice the day after AHR measurement. Cytokines and cells were measured in BAL fluids 24 and 48 hours after the last aerosol challenge. Cells were characterized using the cytospin slides stained with DiffQuick (Baxter Dade AG, Duedingen, Switzerland).

In supernatants of cell cultures, cytokines were assayed using the BioPlex Cytokine Assay according to the manufacturer's recommendations (Bio-Rad, Marnes La Coquette, France).

### 2.7. Airway Hyperresponsiveness (AHR) Measured by Whole-Body Plethysmography 

Whole-body plethysmography was performed in a closed chamber allowing recording the pressure fluctuations during the breathing cycle of mice. “Enhanced pause” (Penh) was calculated as previously described by Hamelmann et al. [12] from the box pressure recorded during inspiration and expiration, and the timing comparison of early and late expiration. Penh corresponds to PEP/PIP, where PEP is peak expiratory pressure and PIP is peak inspiratory pressure. Mice were challenged with pollen by 30 minutes of aerosol (10 mL of 1% pollen extract in 0.9% NaCl) during 3 consecutive days. Pressures were measured 24 hours after the challenge, and Penh values were calculated prior to and during 10 min after aerosol of various doses of methacholine (Sigma-Aldrich, Stonheim, Germany). For each mouse, Penh values were plotted against methacholine concentration (from 0 to 40 mg/mL) and the area under the curve (AUC) was calculated [13].

### 2.8. Statistical Analysis

The GraphPad Prism Software 5.0 (San Diego, CA, USA) was used for statistical analysis (*n* = 10–20 mice per group). Results are expressed as mean ± standard deviation (SD). Antibody responses as well as cell and cytokine data were analyzed using analysis of variance (ANOVA) and Tukey's test for intergroup comparisons. The raw data of Penh values were analyzed using the nonparametric Mann-Whitney *U* test. Penh data were also analyzed using the complete methacholine dose-response curve. For each mouse, Penh was plotted against methacholine concentration (from 0 to 40 mg/mL or from 0 to 10 mg/mL) and the AUC was calculated. Then, data were analyzed using analysis of variance (ANOVA) and Dunnett's test when comparing treated mice with controls and using ANOVA and Tukey's test when comparing all the groups with each other.

## 3. Results

### 3.1. Serological Response to Sensitization and Immunotherapy (Table 1)

At the end of the sensitization period (D0), the detection of serum sIgE, sIgG1, and sIgG2a in all but the control group confirmed the efficacy of the sensitization protocol.

At the end of treatment (D63), sIgE levels remained unchanged in the treated groups but further increased in the Sham group (*P* < 0.001  *versus* EPIT or SLIT). sIgG1 increased similarly with EPIT and SLIT (*P* < 0.05  *versus* Sham). sIgG2a increased only at the end of the treatment with EPIT or SLIT (resp., *P* < 0.001 and *P* < 0.05  *versus* sham). The sIgG2a increase was higher with EPIT than with SLIT (*P* < 0.05).

### 3.2. Cytokines Secreted by Reactivated Spleen Cells after Immunotherapy (Figure 2)

The T-cell response was assessed by measuring the *ex vivo* allergen-specific cytokine production of spleen cells from the 4 groups of mice. Spleen cells reactivated with buffer did not secrete significant amounts of cytokines (data not shown), and, in a preliminary study, the optimal dose was determined by performing a dose-response curve (10, 50, and 100 *μ*g/mL of Phleum pretense extract). Spleen cells from all sensitized mice restimulated with Phl p extract secreted higher amounts of Th2 cytokines (IL-4, IL-5, and IL-10) than those of controls (*P* < 0.05 to *P* < 0.001). Only EPIT was associated with lower levels of INF-*γ* than Controls (*P* < 0.01). As compared with sham, spleen cells from EPIT and SLIT mice secreted lower amounts of IL-4 (resp., *P* < 0.001 and *P* < 0.01) and IL-5 (resp., *P* < 0.01 and *P* < 0.01). EPIT downregulated IL-4 more than SLIT did (*P* < 0.01) and was able to decrease IL-10 (*P* < 0.05* versus* Sham), which was not observed with SLIT. Finally, the IL-4/INF*γ* ratio was lower with EPIT (25 ± 1.3) than with SLIT (30 ± 1.03, *P* < 0.05) or sham (46 ± 3.0, *P* < 0.05). The IL-4/IFN*γ* ratio in the SLIT group was not significantly different from that observed in the sham group.

### 3.3. Immune Cells and Cytokines in Bronchoalveolar Lavage

**Figure 3:**
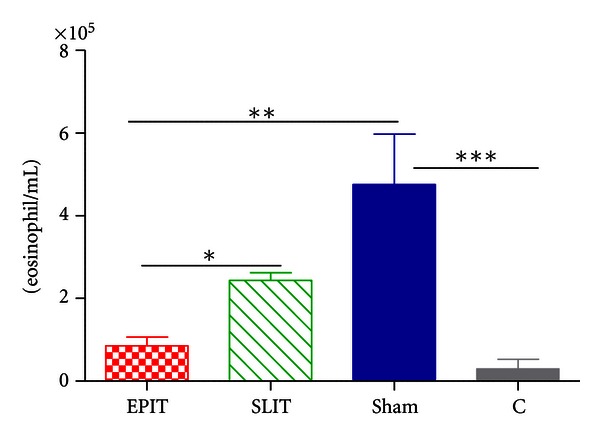
Recruitment of eosinophils in the BAL fluid measured 48 h after the last aerosol challenge. Treatment groups were EPIT (epicutaneous immunotherapy), SLIT (sublingual immunotherapy), sham (sensitized not treated), and C (control, not sensitized and not treated). *P < 0.05, **P < 0.01, ***P < 0.001.

**Figure 4:**
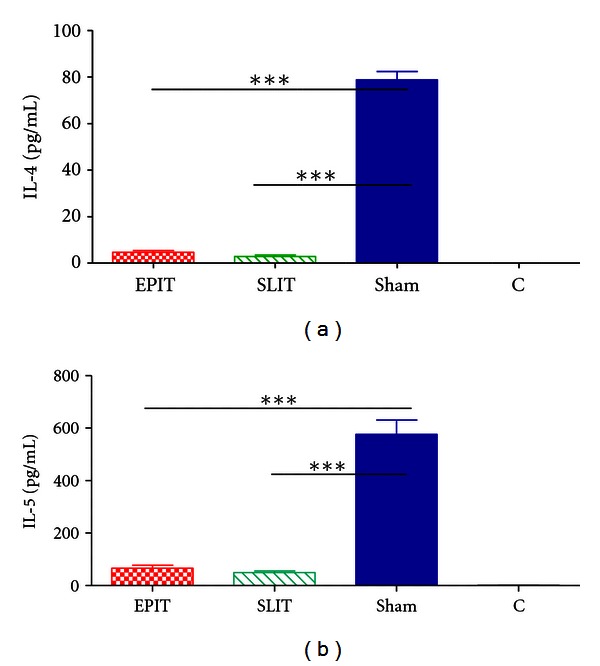
Th2 cytokines (IL-4 (a) and IL-5 (b)) measured in the BAL fluid. After the sensitization and treatment periods, mice were challenged by aerosol during 3 consecutive days. Forty-eight hours after the last challenge, BAL fluids were collected. Treatment groups were EPIT (epicutaneous immunotherapy), SLIT (sublingual immunotherapy), sham (sensitized not treated), and C (control, not sensitized, and not treated). *P < 0.05, **P < 0.01, ***P < 0.001.

EPIT decreased the number of eosinophils in BAL, as compared to sham and SLIT (resp., *P* < 0.01 and *P* < 0.05) (Figure 3). EPIT and SLIT both decreased dramatically IL-4 and IL-5 but not IFN-*γ* (*P* < 0.001, data not shown) in the BAL fluid (Figure 4) and serum (data not shown).

### 3.4. Plethysmography Analysis

**Figure 5:**
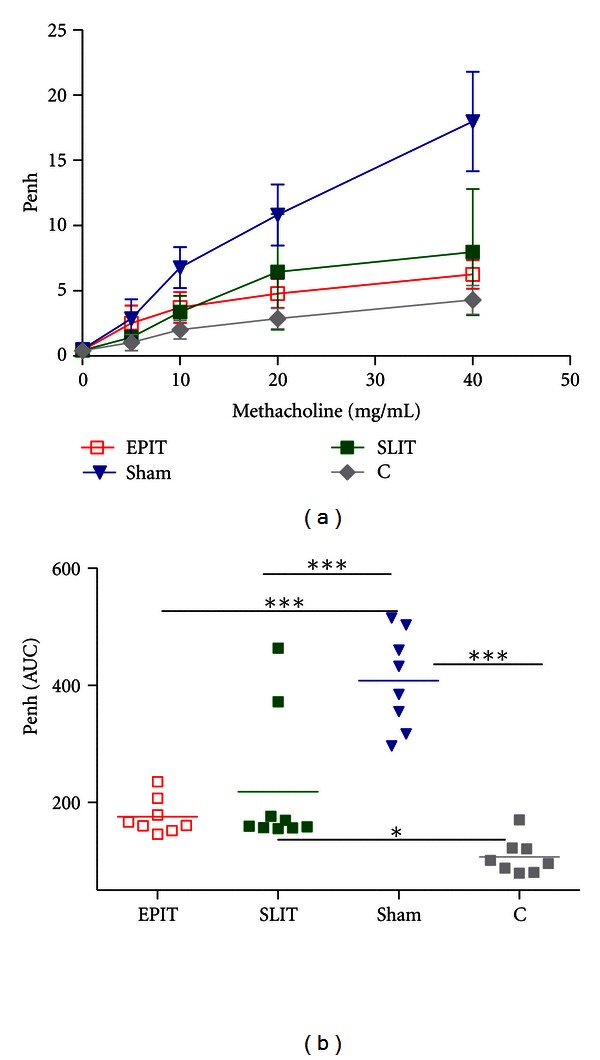
Airway hyperresponsiveness after immunotherapy. Mice were exposed to increasing doses of methacholine the day following the last 30-minute aerosol challenge: (a) dose-response curves and (b) individual area under the curve (AUC) calculated from data of graph (a) Treatment groups were EPIT (epicutaneous immunotherapy), SLIT (sublingual immunotherapy), sham (sensitized not treated), and C (control, not sensitized, and not treated). *P < 0.05, **P < 0.01, ***P < 0.001.

The airway hyperresponsiveness (AHR) to methacholine exposure was measured by plethysmography after 3 days of aerosol challenge. The sham group responded to increasing doses of methacholine with marked AHR; EPIT and SLIT significantly decreased AHR (Figure 5(a)). At the highest dose of methacholine (40 mg/mL), mean Penh values for Sham, EPIT, SLIT, and controls were, respectively, at 17.9 ± 3.8, 6.2 ± 1.1 (*P* < 0.001* versus* sham), 7.9 ± 4.8 (*P* < 0.001), and 4.3 ± 1.1 (*P* < 0.001). EPIT and SLIT also decreased Penh AUC values, as compared with sham (*P* < 0.001) (Figure 5(b)). Interestingly, EPIT induced a homogenous AHR decrease in all mice. With SLIT, 2 mice did not respond to treatment and did not improve AHR. As a result, whereas AUC values following EPIT were identical to those of control mice, those following SLIT remained higher (*P* < 0.05).

## 4. Discussion

These data confirm the results obtained in previous studies using the same protocol in sensitized mice treated with comparable efficacy with EPIT or SCIT [9].

 In the present study, validated methods of sensitization were used [6, 14, 15]. This was here confirmed by the detection of sIgE, sIgG1, and sIgG2 in each sensitized group, as previously reported with the same protocol [9]. Increased Penh values at every dosage of methacholine in the sham group confirmed the accuracy of this model of bronchial hyperresponsiveness and were consistent with the literature [12].

Due to the high levels of specific IgG1 and to verify that the ELISA method used did not underestimate specific IgE levels, a reverse enzyme allergosorbent assay was performed in which total serum IgE antibodies were first captured by immobilized anti-mouse IgE monoclonal antibodies [9]. Results of this experiment performed after EPIT in peanut-sensitized mice were comparable to those of ELISA.

To date, the *in vivo *measurement of respiratory function in mice is based on both noninvasive and invasive approaches. Whole body plethysmography is noninvasive and has been used in various mouse models of allergy [16, 17]. It allows recording the pressure fluctuations that occur during the breathing cycle of mice and measures a single parameter called Penh. In a previous study comparing EPIT and SCIT in mice sensitized to peanuts, we performed both whole body plethysmography and intratracheal resistance/compliance measurements. Results appeared perfectly similar with the two methods of investigation [18]. Hence, for ethical reasons, only plethysmography was here evaluated.

EPIT was performed using Viaskin, a new epicutaneous delivery system (EDS) promoting, without any adjuvant, dissemination of allergens in the thickness of the stratum corneum, in contact with the immune cells of the epidermis [19]. By contrast with previous studies, the use of Viaskin allowed performing EPIT without requiring any skin stripping [20–22]. We have already demonstrated the efficacy of this model of EPIT with regard to four clinically relevant allergens, 2 respiratory allergens (*Dactylis glomerata* pollen and house dust mite) and 2 food allergens (peanut and ovalbumin) [9]. The current study thus extends the application of EPIT using Viaskin to another allergy to pollen (i.e.,* Phleum pratense*) and clearly states the equivalent efficacy of EPIT to SLIT method.

For SLIT, despite extensive investigations in clinical practice [23], very few animal models have been published. The practical difficulties of developing a suitable animal model probably explain the low number of studies in this area. The only two studies comparable to the present one, and published to date used a different procedure for the sublingual administration of the allergen. To prevent animals from swallowing the allergen solution, Brimnes et al. [6] were holding them by the scruff of the neck during 20 seconds after application of the allergen under the tongue. In the other study, the allergen was absorbed in a mucoadhesive solution, which was fixed under the tongue [24]. In the current study, the viscosity of the solution was increased using CMC in addition to pollen proteins and every sublingual administration of Ph1p was performed under general anesthesia during a long period (from 30 to 60 minutes). As general anesthesia depresses swallowing [25], it ensures optimal contact of the mucosa with allergen. Using Evans Blue dye combined with CMC, dye appeared to be only detectable in sublingual tissues until 30 minutes after sublingual treatment contrary to the model of Brimnes et al. [6], where dye was also found inside the stomach. The immune response to the sublingual treatment, globally similar to that of other studies, and in some points to EPIT in the current study, confirms the efficacy of this route of administration.

EPIT and SLIT were both overall efficient in this study but slight differences were observed according to the technique used. The dramatic increase in sIgE levels observed during the 8 weeks of sham treatment in sham was equally suppressed by EPIT and by SLIT. In accordance with our previous results [9], EPIT and SLIT increased IgG2a, the mice equivalent of humans IgG4, but this increase was significantly higher with EPIT than with SLIT. This serological response compares with the clinical data since it is well established that SLIT treatment leads to a systemic increase of sIgG4 whereas sIgEs increase or remain stable [26].

EPIT decreased T_H_2-related cytokines (IL-4 and IL-5) in serum and BAL, as well as the IL-4/INF-*γ* ratio in spleen cell culture supernatant. Overall, these changes reflect the switch of the immune response from a T_H_2 to a balanced T_H_2/T_H_1 profile. However, as compared to SLIT, EPIT seemed to induce a stronger reduction of the T_H_2 response with a sharper decrease of IL-4, IL-5, IL-10, and IL-13, a trend of increase of TGF-*β* (not significant), and, in the bronchoalveolar lavage, a dramatic decrease of eosinophils.

EPIT and SLIT decreased the Penh values in the same proportions, from the lowest dose of methacholine (10 mg/mL to the highest 40 mg/mL). However, if EPIT improved Penh in all mice, SLIT did not in 2 mice. This resulted in the fact that Penh AUC values after EPIT returned to values similar to these of control mice, which was not seen with SLIT (*P* < 0.05* versus* controls).

In this study, EPIT and SLIT were given once a week at the same dose, that is, 100 *μ*g of pollen proteins, containing 5 *μ*g of the major allergen Phlp5, the effective dose described by Brimnes et al. in their murine model [6]. Anesthesia probably allowed better contact of allergen with the mucosa, by inhibiting swallowing. The efficacy of SLIT for all the study parameters appeared to be better than that previously published by Brimnes et al. [6], despite lower dose and frequency of administration.

The mechanistic reasons why EPIT and SLIT exhibited differences in the different studied parameters lack clarity. Whereas EPIT specifically targets the immune cells of the superficial layers of the skin, particularly skin Langerhans cells (LCs), SLIT targets dendritic cells (DCs) of the oral mucosa. We have demonstrated in another study with ovalbumin conjugated to fluorochrome that the allergen was detected in the superficial layers of the skin. Of note is that, within the first 24 hours following application, the allergen was only found in the antigen presenting cells, and more particularly in more than 90% of the LC of the epidermis and almost 50% of the dermal DC of the dermis [19]. After having captured allergen following epicutaneous or sublingual administration, DCs migrate to draining lymph nodes and stimulate lymphocytes. As suggested in studies by Strid et al., these cells may play a key role by influencing the immune profile of the reaction [20–22]. We have demonstrated that EPIT may be able to strongly influence both the migration of LC to the lymph node and lymphocytes stimulation [19]. Even if classic skin and oral LC show similar kinetics of migration to lymph nodes, they differ phenotypically and functionally [27]. In addition, the number of DC migrating from the skin is higher than from the buccal mucosa [28]. These data may explain part of the differences observed between EPIT and SLIT.

Senti et al. [7] recently described the epicutaneous administration of specific grass pollen allergens in patients with seasonal rhinitis. This pilot study suggested that immunotherapy could occur successfully via the skin route with repeated and prolonged applications of the allergen to the skin. A pilot study was also conducted in children with IgE-mediated cow's milk allergy [8]. As the method used in that clinical study does not need any skin preparation, by contrast to that described by Senti et al. [7], it is our experience that it was very well tolerated in children.

## 5. Conclusions

In conclusion, these data suggest that the epicutaneous route might be at least as potent as the sublingual route for immunotherapy in an animal model. The immune mechanisms involved in this therapeutic process need further investigation but these data confirm that EPIT may represent a promising new route for immunotherapy, alternative to the sublingual route of administration.

## Figures and Tables

**Figure 1:**
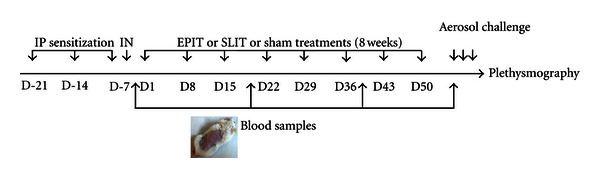
Study design. Mice were sensitized to pollen proteins by 3 subcutaneous injections, with aluminium hydroxide as adjuvant, separated at 1-week interval. Immunotherapy was conducted during 8 weeks with one treatment per week: one sublingual administration or one 48-h application of Viaskin epicutaneous delivery system. Blood was sampled before immunotherapy (D0) to validate the phase of sensitization and during immunotherapy (D21, D38, D63).

**Figure 2:**
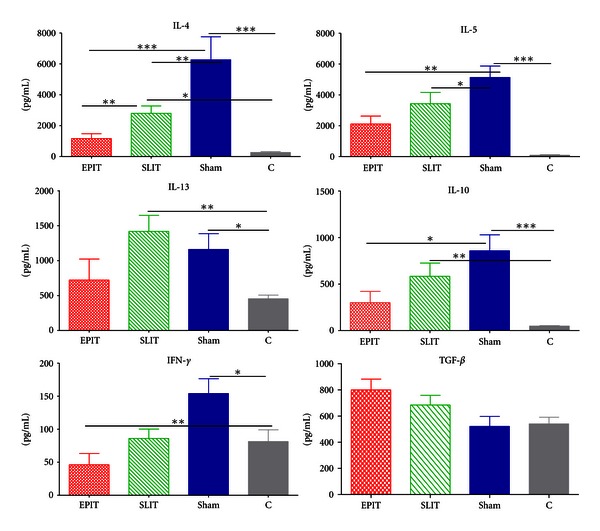
Ex vivo cytokine production of spleen cells restimulated with Phl p extract (100 *μ*g/mL). Treatment groups were EPIT (epicutaneous immunotherapy), SLIT (sublingual immunotherapy), sham (sensitized not treated), and C (control, not sensitized, and not treated). *P < 0.05, **P < 0.01, ***P < 0.001.

**Table 1:** Quantification of specific IgE (ng/mL), IgG1, and IgG2a (*μ*g/mL) for EPIT, SLIT, sham, and Control (C) groups.

Pollen-specific antibodies		EPIT	SLIT	Sham	C
IgE	**D0**	57 (±9.8)	48 (±1.7)	84 (±10.5)	Und
	**D21**	86 (±8.7)	88 (±10.4)	79 (±7.1)	Und
	**D38**	55 (±1.7)	138 (±43.4)	88 (±12.4)	Und
	**D63**	71 (±9.0)***	58 (±4.5)***	137 (±11.9)	Und

IgG1	**D0**	1.1 (±0.15)	1.1 (±0.1)	1.3 (±0.2)	Und
	**D21**	18.2 (±1.36)	10.6 (±0.8)	7.7 (±1.3)	Und
	**D38**	57.1 (±3.6)	37.5 (±4.9)	10.3 (±2.1)	Und
	**D63**	113.6 (±13.2)***	75.8 (±20.2)***	18.0 (±3.6)	Und

IgG2a	**D0**	0.06 (±0.001)	0.01 (±0.002)	0.02 (±0.002)	Und
	**D21**	0.03 (± 0.006)	0.05 (± 0.011)	0.05 (± 0.032)	Und
	**D38**	0.03 (± 0.012)	0.08 (± 0.042)	0.03 (± 0.011)	Und
	**D63**	0.14 (± 0.008)^†^,***	0.09 (± 0.031)*	0.001 (± 0.001)	Und

Statistical comparison, EPIT *versus* SLIT: ^†^ *P* < 0.05.

Statistical comparison, EPIT, or SLIT *versus* Sham: *P < 0.05, **P < 0.01, ***P < 0.001.

C, control; EPIT: epicutaneous immunotherapy; IgE: immunoglobulin E; IgG1: immunoglobulin G1; IgG2a: immunoglobulin G2a; SLIT: sublingual immunotherapy; Und: undetectable.

## Abbreviations

AHR: Airway hyperresponsiveness
BAL: Bronchoalveolar lavage
CMC: Carboxymethyl cellulose
DC: Dendritic cells
EDS: Epicutaneous delivery system
EPIT: EPicutaneous immunotherapy
LC: Langerhans cells
Penh: Enhanced pause
SLIT: SubLingual immunotherapy.
